# A variant of the venom allergen-like protein, *Dd*VAP2, is required for the migratory endoparasitic plant nematode *Ditylenchus destructor* parasitism of plants

**DOI:** 10.3389/fpls.2023.1322902

**Published:** 2023-12-13

**Authors:** Qing Chang, Yiwei Yang, Bo Hong, Yanqun Zhao, Mengxin Zhao, Shanshan Han, Feng Zhang, Huan Peng, Deliang Peng, Yingmei Li

**Affiliations:** ^1^ Shaanxi Key Laboratory of Plant Nematology, Bio-Agriculture Institute of Shaanxi, Xi’an, China; ^2^ Yulin Agricultural Technology Service Center, Yulin, China; ^3^ State Key Laboratory for Biology of Plant Diseases and Insect Pests, Institute of Plant Protection, Chinese Academy of Agricultural Sciences, Beijing, China

**Keywords:** migratory endoparasitic plant nematode, *Ditylenchus destructor*, effector, venom allergen-like protein, RNAi

## Abstract

The potato rot nematode, *Ditylenchus destructor*, poses a serious threat to numerous root and tuber crops, yet the functional characterization of effectors from this migratory endoparasitic plant nematode remains limited. Despite inhabiting distinct habitats, sedentary and migratory plant parasitic nematodes share the structurally conserved effectors, such as venom allergen-like proteins (VAPs). In this study, a variant of *DdVAP2* was cloned from *D. destructor*. The transcription profile analysis revealed that *DdVAP2* was higher expressed in *D. destructor* feeding on either potato or sweet potato compared to on fungus via qRT-PCR. And *DdVAP2* was highly expressed at all life stages feeding on sweet potato, except for eggs. *Dd*VAP2 was confirmed to be specifically expressed in the subventral esophageal glands of *D. destructor* through *in situ* hybridization assays. Combined with functional validation of the signal peptide of *Dd*VAP2, it suggested that *Dd*VAP2 could be secreted from nematode into host. Heterologous expression of *Dd*VAP2 in *Nicotiana benthamiana* revealed that the protein localized in both cytosol and nuclei of plant cells. Knocking down *DdVAP2* by RNAi in *D. destructor* resulted in infection and reproduction defects on plants. All the results suggest that *DdVAP2* plays a crucial role in the interaction between *D. destructor* and plants by facilitating the nematode infection.

## 1 Introduction

Potato rot nematode, *Ditylenchus destructor* Thorne, 1945, is one of the most serious pathogens on sweet potato (Xu et al., 2015), and the second most serious plant-parasitic nematode on potato (Faulkner and Darling, 1961; Plowright et al., 2002; Pridannikov, 2021). In general, the yield losses caused by *D. destructor* could be 20% to 50%, and even 100% in endemic regions. In addition, *D. destructor* can infect more than 120 plant species, as well as feed and reproduce on approximately 70 fungal species, such as *Alternaria*, *Botrytis*, *Fusarium*, and *Penicillium* genera (Sturhan and Brzeski, 1991). Due to its serious harm and persistence once established in fields, *D. destructor* has been listed as a significant quarantine pest in many countries (Eppo, 2017). Despite being a destructive plant parasitic nematode threatening food security worldwide, effective disease control measures for *D. destructor* remain limited (Abd-Elgawad, 2022). To develop new control strategies, it is urgent to uncover its pathogenic mechanism.

Plant parasitic nematodes can secrete proteins or molecules through their stylet or body wall to facilitate their invasions, migrations, inducing and maintaining feeding sites within host plants (Vieira and Gleason, 2019). These secretory proteins, known as effectors, can ascend nematodes’ parasitism by degrading and modifying plant cell walls, inhibiting defense responses, and disturbing host signaling pathways (Ali et al., 2017; Mejias et al., 2019; Goverse and Mitchum, 2022). Therefore, cloning and functional characterizing effectors is the key to understand the molecular basis of plant nematode pathogenic mechanisms (Eves-Van Den Akker et al., 2021). However, most of our knowledge on plant nematode effectors comes from sedentary endoparasitic nematodes, such as root-knot nematodes and cyst nematodes (Pogorelko et al., 2020; Jagdale et al., 2021; Rutter et al., 2022). Being different life style from sedentary endoparasitic nematodes, *D. destructor* is a migratory endoparasitic nematode that does not induce a feeding structure and always migrates within the host plants (Zheng et al., 2016). Therefore, comprehensive studies on its own effectors are necessary to reveal its unique parasitic mechanism (Mathew and Opperman, 2020). Although homologues of many effectors identified from sedentary plant parasitic nematodes have also been found in *D. destructor* (Peng et al., 2013), further functional research on these effectors is very limited. Only β-1,4-endoglucanases, alpha-amylase genes and several subunit genes of the voltage-gated calcium channel had been functionally characterized in *D. destructor* to date (Peng et al., 2014; Chen et al., 2020; An et al., 2022; Chen et al., 2022).

Venom allergen-like proteins (VAPs) are highly conserved with cysteine residues at the carboxyl terminus, belonging to the cysteine-rich secretory proteins/antigen 5/pathogenesis-related 1 (CAP) protein superfamily (Wilbers et al., 2018). They are ubiquitously present not only in plants and animals, but also in nematodes with diverse ecological niches. Since the first nematode VAP, Ac-ASP- 1, was identified from *Ancylostoma caninum* (Hawdon et al., 1996), VAPs had been found not only in *A. caninum* (Hawdon et al., 1999), but also in various animal parasitic nematodes, such as *Haemonchus contortus*, *Schistosoma mansoni* and *Brugia malayi* (Schallig et al., 1997; Chalmers et al., 2008; Darwiche et al., 2018), as well as the free-living model nematode, *Caenorhabditis elegans* (Mohandas et al., 2015). In plant parasitic nematodes, there were a few to a lot of VAPs widely present. For cyst nematodes, 2 VAPs were identified from both *Heterodera glycines* and *Heterodera avenae* (Gao et al., 2001; Luo et al., 2019). In addition, 8, 8 and 10 VAPs were identified from *Globodera pallida*, *Heterodera schachtii* and *Globodera rostochiensis*, respectively (Van Steenbrugge et al., 2022). In *Meloidogyne* spp., several VAPs were identified from different root-knot nematodes, such as *M. incognita*, *M. hispanica*, *M. arenaria* and *M. graminicola* (Ding et al., 2000; Wang et al., 2007; Duarte et al., 2014; Petitot et al., 2020). Furthermore, VAPs were also found in different migratory plant parasitic nematodes, such as *Bursaphelenchus xylophilus*, *Bursaphelenchus mucronatu* and *Ditylenchus africanus* (Haegeman et al., 2009; Yan et al., 2012). Especially in *B. xylophilus*, a cluster of three venom allergen-like protein genes and one pseudogene were identified (Lin et al., 2011). Although 2 VAPs was identified in *D. destructor* (Peng et al., 2013), no further functional research had been conducted on these *Dd*VAPs so far. As a kind of highly conserved effectors in different nematodes, the biological roles and functions of different VAPs deserve further investigations.

Early functional studies of VAPs confirmed that VAPs could be secreted into plants in the interaction between either sedentary or migratory plant parasitic nematodes and hosts (Gao et al., 2001; Yan et al., 2012). In *B. xylophilus*, the stage-specific transcription analysis demonstrated that two of the three VAPs were highly expressed in the nematodes interacting with plants, and knockdown of *Bxvap-1* by RNAi reduced the nematode migration in pinewood (Kang et al., 2009; Kang et al., 2012). Similarly, up-regulation of *Mi-vap-2* in *M. incognita* enhanced the nematode pathogenicity to *Nicotiana benthamiana* (Chi et al., 2016). In the study of a migratory plant parasitic nematode, *Radopholus similis*, abundant expression of *RsVAP* in juveniles facilitated the nematode infection of plants (Li et al., 2021). Furthermore, some studies revealed that plant parasitic nematode VAPs can also act as both activators and suppressors of plant defense responses. For instance, Gr-VAP1, an VAP from *G. rostochiensis*, was shown to be able to trigger a defense response mediated by the surface-localized immune receptor Cf-2 in tomato, once Gr-VAP1 interacted with the apoplastic cysteine protease Rcr3^pim^ of tomato (Lozano-Torres et al., 2012). Whereas, in the absence of Cf-2, Gr-VAP1 could promote nematode virulence by binding to Rcr3^pim^. Moreover, knockdown of Gr-VAP1 reduced the nematode virulence, and ectopic expression of Gr-VAP1 increased the potato susceptibility to *G. rostochiensis* (Lozano-Torres et al., 2014). Combined with the enhanced susceptibility of *Arabidopsis thaliana* to *Heterodera schachtii* with ectopic expression of either Gr-VAP1 or its homologous in *H. schachtii*, Hs-VAP1 or Hs-VAP2, it could be concluded that the VAPs from different cyst nematodes could commonly enhance the susceptibility of different plants to cyst nematodes (Lozano-Torres et al., 2014). While in *H. avenae*, two VAPs showed distinct functions in its parasitism (Luo et al., 2019). Knockdown of *HaVAP1* increased the nematode virulence, while silencing of *HaVAP2* hampered the parasitism of the nematode. Although the promoting and inhibiting effects of VAPs had both been reported in the parasitism of different phytophagous nematodes, the function of VAPs in the interaction between *D. destructor* and plants remains elusive.

In this study, the encoding gene of an venom allergen-like protein, *DdVAP2*, was cloned from *D. destructor* based on the EST library (Peng et al., 2013). With a signal peptide predicted from *Dd*VAP2 by bioinformatics analysis, its secretion function was validated in the yeast system. The transcript profiles of *DdVAP2* in mixed life stage *D. destructor* populations feeding on potato, sweet potato and *Fusarium semitectum* were compared by qRT-PCR. Additionally, the expression profile of *DdVAP2* in different life stages of *D. destructor* was further analyzed. To further understand the tissue distribution of *DdVAP2* in nematode and localization of *Dd*VAP2 protein in plants, *in situ* hybridization and subcellular localization assays were conducted, respectively. Finally, the function of *DdVAP2* in *D. destructor* parasitism was analyzed by knockdown its expression with RNAi experiments. All the results indicated the important role of *DdVAP2* in ascending the nematode infection during the interaction between *D. destructor* and plant, shedding light on the functions of VAP in the parasitism of this serious migratory endoparasitic plant nematode.

## 2 Materials and methods

### 2.1 Nematode culture and collection

The *D. destructor* isolate was extracted from infected sweet potatoes in Xi’an, Shaanxi, China using the modified Baermann funnel method (Viglierchio and Schmitt, 1983). After soaked in 0.3% streptomycin sulfate for 30 min for surface sterilized, the nematodes were washed with sterile water for three times. Then the nematodes were maintained on plates of *F. semitectum* cultured on potato dextrose agar (PDA) medium at 26°C, as described previously (Peng et al., 2014). Nematodes in mixed life stages were collected from the plates with distilled water after 30 d culture in the dark. Nematode eggs were collected by density gradient centrifugation with 70% (m:v) solutions of sucrose, and J2s were molted from the collected eggs. Males and females were manually separated under an SZX16 stereomicroscope (Olympus, Tokyo, Japan).

For infection assays, potatoes (cv. Longshu no. 7) and sweet potatoes (cv. Yanshu no. 25) were thoroughly washed with clean water, sterilized with 1% NaClO for 10 min, dried in clean bench and treated with UV light for 30 min for surface sterilization prior to inoculation. Sterilized nematodes with mixed stages were then inoculated into the potatoes or sweet potatoes by digging holes with a sterilized scalpel. After the holes filled with excavated tissues from the holes, the inoculated potatoes or sweet potatoes were sealed with paraffin and incubated at 26°C in the dark. After 30 d of incubation, the nematodes in mixed life stages were collected from the potatoes or sweet potatoes using the modified Baermann funnel method as mentioned above.

### 2.2 Cloning and phylogenetic analysis of *DdVAP2*

Total RNA was extracted from the mixed life stages of *D. destructor* collected from sweet potatoes using TRIzol reagent (Invitrogen, New York, USA), and then reverse transcribed into cDNA using the FastKing One Step RT-PCR kit (Tiangen, Beijing, China). The open reading frame (ORF) of *DdVAP2* was cloned from the cDNA using the primers FL-F/FL-R (**Table S1**) based on the sequence of *Dd*VAP2 (GenBank accession no. GU370352). The amplified product was sequenced after cloned into pMD19-T vector (Takara, Tokyo, Japan). The resulting sequence was identified by BLAST analysis. The effects of different amino acid substitutions were analyzed using SFIT4G (https://sift.bii.a-star.edu.sg/sift4g/AnnotateVariants.html). The domains and active sites were predicted with the deduced protein sequence using InterProScan on the InterPro website (https://www.ebi.ac.uk/interpro/). The protein molecular weight, theoretical pI and amino acid composition were predicted using the ProtParam tool on the Expasy website (https://web.expasy.org/protparam/). The signal peptide of *Dd*VAP2 was predicted using both SignalP 5.0 (https://services.healthtech.dtu.dk/services/SignalP-5.0) and Phobius (https://phobius.sbc.su.se). All the VAPs identified from different plant parasitic nematodes were obtained with their GenBank numbers. Homologues and paralogues of *Dd*VAP2 from different *D. destructor* isolates were obtained in the non-redundant protein database using BLASTp. Then, all the protein sequences obtained were used for the construction of a phylogenetic tree using MEGA 6.0.

### 2.3 Functional validation of the signal peptide

As the first 21 amino acids of *Dd*VAP2 were predicted as a signal peptide by both SignalP 5.0 and Phobius, the first 63 nucleotide acids of *DdVAP2* were cloned with the primers SP-F/SP-R (**Table S1**). Then the sequence encoding the signal peptide of *Dd*VAP2 was cloned into the vector pSUC2 for functional validation in the yeast strain YTK12. The encoding sequence of the Avr1b signal peptide and the sequence encoding the first 25 amino acids of Mg87 were also cloned into the vector pSUC2 for serving as positive and negative controls, respectively. Then the empty vector pSUC2 and the three recombinant vectors, DdVAP2::pSUC2, Avr1b::pSUC2, Mg87::pSUC2, were transformed into YTK12 cells using the lithium acetate method, respectively, as described previously (Chang et al., 2017). The transformants were isolated from the CMD-W medium (0.67% yeast nitrogen base without amino acids, 0.075% tryptophan dropout supplement, 2% sucrose, 0.1% glucose and 2% agar), and inoculated on the YPRAA medium (1% yeast extract, 2% peptone, 2% raffinose, 2 mg/ml antimycin A and 2% agar) at equivalent concentrations.

### 2.4 Transcription profile of *DdVAP2* in *D. destructor*

Total RNAs were extracted from the mixed life stages nematodes cultured on *F. semitectum*, potatoes or sweet potatoes as mentioned above, respectively. To analyze the transcription levels of *DdVAP2* in different life stages, approximately 2,000 individuals of eggs, J2s, males and females were collected for RNA extraction from the population cultured on sweet potato, respectively. The RNA concentration of each sample was determined using a NanoDrop 2000 spectrophotometer (Thermo Scientific, Massachusetts, USA), and then the RNA was reverse transcribed into cDNA for each sample using 1µg of RNA. Then the qRT-PCR was performed with TB Green Premix Ex Taq (Takara, Tokyo, Japan) in a Bio-Rad CFX96 Cycler (Bio-Rad, California, USA) to quantify the transcription level of *DdVAP2* in each sample. With actin gene (GenBank accession no. GQ327968) served as the internal reference gene, relative transcript levels of *DdVAP2* were calculated using the 2^-ΔΔCt^ method (Peng et al., 2014). All the primers used are listed in **Table S1**. Three independent replicates were conducted for each sample, and the entire experiment was repeated with two independent biological replicates. Differences were considered significant at a probability level of P<0.05.

### 2.5 *In situ* hybridization of *Dd*VAP2 in *D. destructor*

For *in situ* hybridization, two fragments from the ORF of *DdVAP2* selected as probes were amplified using the primers Probe1-F/Probe1-R and Probe2-F/Probe2-R (**Table S1**). The sense and antisense probes were labeled with digoxigenin (DIG) using the PCR DIG Probe Synthesis Kit (Roche, Basel, Switzerland) with the PCR products. The nematodes were fixed in 2% paraformaldehyde at 4°C for 48 h and an additional 6 h at room temperature, then cut and digested with 0.5 mg/mL proteinase K at room temperature for 1 h, subsequently. The hybridization was performed as described by de Boer et al. (De Boer et al., 1998). Hybridization signals were detected using the DIG High Prime DNA Labeling and Detection Starter Kit II (Roche, Basel, Switzerland). The nematodes were observed and photographed under an Olympus BX53 microscope (Olympus, Tokyo, Japan).

### 2.6 Subcellular localization of *Dd*VAP2 in *N. benthamiana*

To clarify the subcellular localization of *Dd*VAP2 in plants, the ORF of *DdVAP2* without the signal peptide and terminator was cloned into the *N. benthamiana* expression vector pCAMBIA1302 using the primers SL-F/SL-R (**Table S1**). The plasmids of the original vector pCAMBIA1302 and the DdVAP2ΔSP::pCAMBIA1302 were transformed into *Agrobacterium tumefaciens* GV3101. After overnight culture, *A. tumefaciens* cells were precipitated and re-suspended in the infiltration buffer as previously described (Luo et al., 2019). The suspensions were kept at room temperature for 3 h in the dark before being infiltrated into four-week-old *N. benthamiana* plants. Infiltrated leaves were sampled and observed using a Zeiss LSM 510 META confocal laser scanning microscope (Zeiss, Jena, Germany). Green fluorescence was observed with excitation at 488 nm and emission at 510 nm.

### 2.7 RNAi of *DdVAP2* and pathogenicity assay

Suitable fragments for RNAi of *DdVAP2* were identified by predicting potential RNAi targets from the ORF of *DdVAP2* using siDirect (https://www.sidirect2.rnai.jp/) and DSIR (https://www.biodev.extra.cea.fr/DSIR/DSIR.html). Two target fragments (RNAi-1 and RNAi-2) were obtained using the primers RNAi-1F/RNAi-1R and RNAi-2F/RNAi-2R, respectively (**Table S1**). The fragment of GFP (GenBank accession no. HF67500) was used as a negative control. The dsRNA were synthesized from the three fragments using the HiScribe T7 Quick High Yield RNA Synthesis Kit (NEB, Massachusetts, USA). After purifying the dsRNA with the MEGAclear Kit (Ambion, Texas, USA), nematodes were incubated with the soaking buffer containing the dsRNA of RNAi-1, RNAi-2 and GFP, respectively, as described previously (Peng et al., 2014). The treatment group, in which nematodes were incubated in the soaking buffer without any dsRNA, served as a mock control. Fluorescein isothiocyanate isomer-1 (FITC) was used to track the ingestion of dsRNA by nematodes, as described previously (Peng et al., 2014). Approximately 1,000 individuals of *D. destructor* were collected after incubation for 24, 48 and 72 h to determine the optimal soaking period for RNAi of *DdVAP2*. The RNA extraction from the nematodes of each treatments with different incubation times, reverse transcription into cDNA and qRT-PCR was performed as mentioned above. Two biological replicates were performed for each soaking time.

To assess pathogenicity, approximately 500 individuals of different treatments (RNAi-1, RNAi-2, GFP and mock) were inoculated to sweet potato seedlings as described previously (Peng et al., 2014). Roots were collected and stained using acid fuchsin at 7 days post inoculation (dpi), and nematodes within roots were counted under the microscope. In addition, approximately 500 individuals of different treatments (RNAi-1, RNAi-2, GFP and mock) were inoculated into sweet potato storage roots. After 20 days, all the nematodes in different storage roots were collected using the modified Baermann funnel method and counted. Then the reproduction rates were calculated as previously described (Ye et al., 2020).

Reproduction rate = Final numbers of nematodes/Initial numbers of nematodes.

All infection assays were performed in five individual seedlings or storage roots. And two biological replicates were performed. Statistical analysis was performed with one-way ANOVA using SPSS 14.0. Differences between groups were considered significant at a probability level of P<0.05.

## 3 Results

### 3.1 Cloning, sequencing and phylogenetic analysis of *DdVAP2*

**Figure 1:**
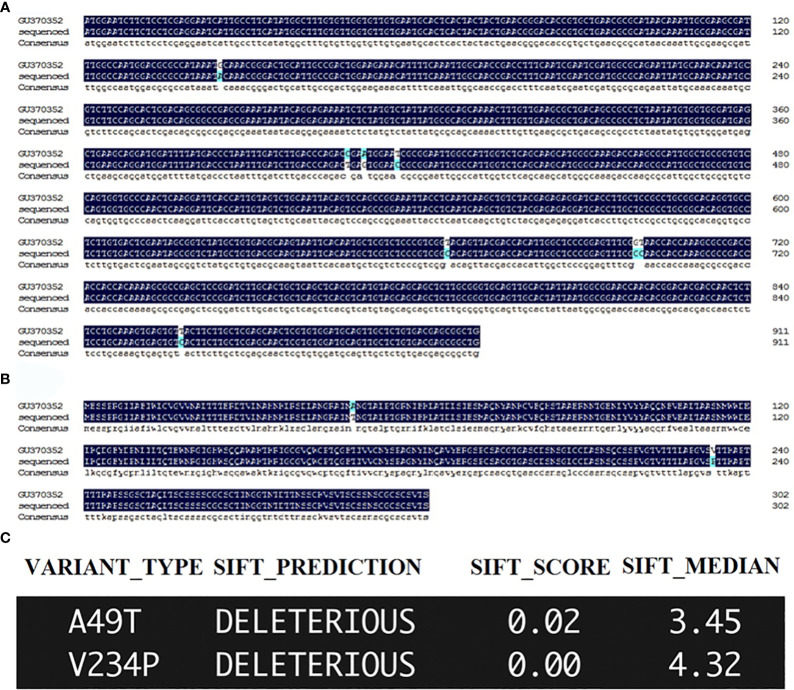
Sequencing and sorting intolerant from tolerant (SIFT) analysis of the sequenced *DdVAP2*. **(A)** ORF alignment of the sequence obtained and sequenced in this study with the published *DdVAP2* (GU370352). GU370352: the sequence published on NCBI by a former study; sequenced: the sequence obtained and sequenced in this study. **(B)** Alignment of the amino acid sequences encoded by the *DdVAP2* sequenced in this study and the published *DdVAP2* (GU370352). GU370352: the sequence published on NCBI by a former study; sequenced: the sequence obtained and sequenced in this study. **(C)** The effects of the two amino acid substitutions between the two amino acid sequences of *DdVAP2* were analyzed using SFIT4G (https://sift.bii.a-star.edu.sg/sift4g/AnnotateVariants.html).

The orthologue of *DdVAP2* (GenBank accession no. GU370352) was cloned from *D. destructor* isolated in Xi’an, Shaanxi, China. In comparison with the original sequence published on NCBI (GenBank accession no. GU370352), 8 nucleotide substitutions were found among the total 912 nucleotides that encode the ORF of *DdVAP2*, resulting in 2 amino acid substitutions in the protein sequence, in the cloned and sequenced *DdVAP2* (GenBank accession no. OR523227) in this study (**Figure 1**). SIFT analysis suggested that these substitutions might be deleterious to the protein function (**Figure 1C**). We speculated that these changes could be a result of single nucleotide polymorphisms (SNPs) between different isolates of *D. destructor*, and the sequence we cloned could be a new variant of *DdVAP2*. For all functional analysis conducted in this study, we used this new variant that still named as *DdVAP2*.

**Figure 2:**
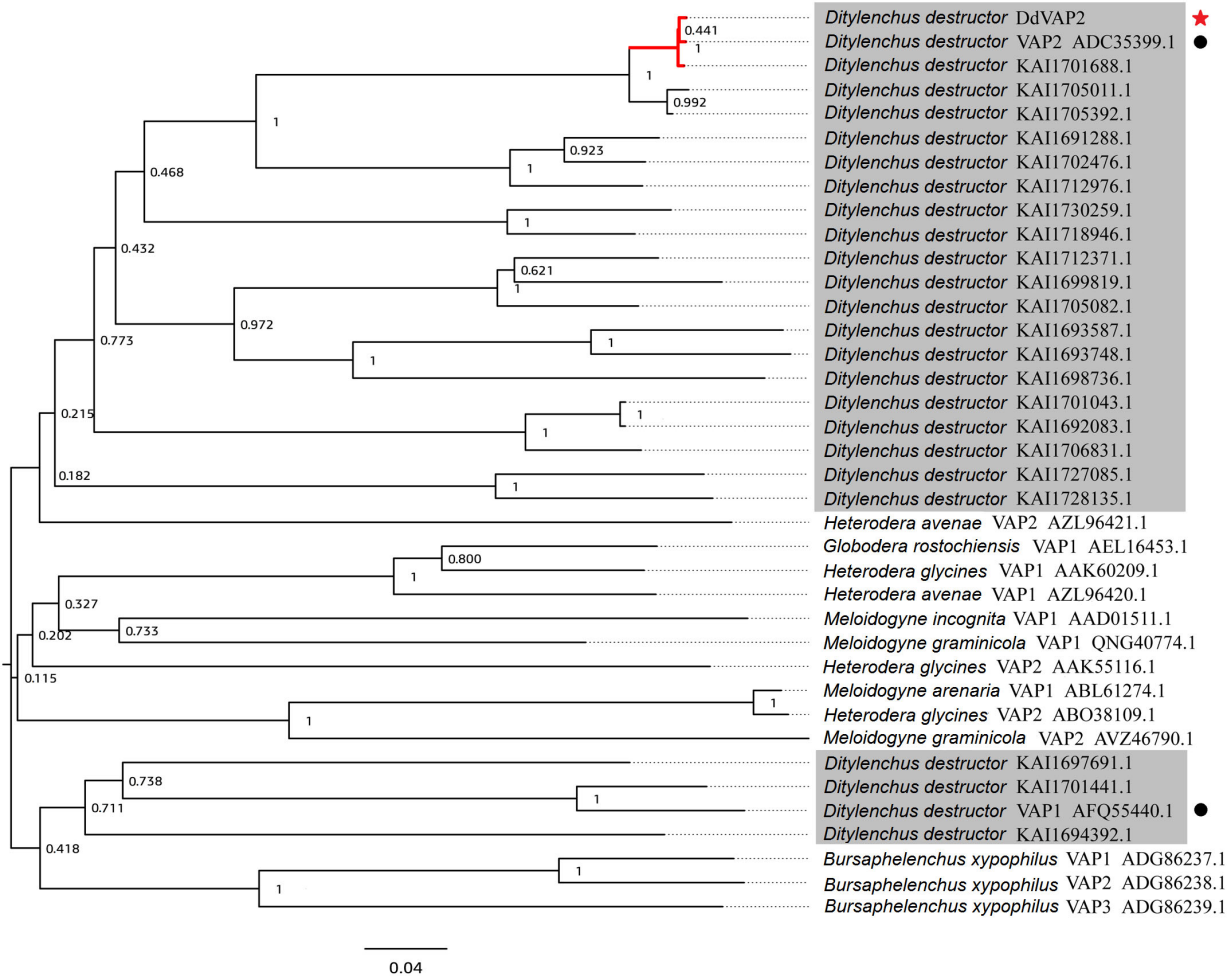
Phylogenetic analysis of VAPs from *Ditylenchus destructor* and other plant parasitic nematodes. All the shaded sequences are from *Ditylenchus destructor*. Black circles indicate two VAPs cloned from *Ditylenchus destructor* before, and the red star indicate the *Dd*VAP2 cloned and sequenced in this study.

The deduced *Dd*VAP2 protein is composed of 303 amino acids, with a calculated molecular weight of 31.9 kDa and a theoretical isoelectric point (pI) of 6.76. Both SignalP 5.0 and Phobius analysis predicted a signal peptide comprised of the first 21 amino acids in *Dd*VAP2 (**Figure S1**), suggesting that this protein could be secreted. InterProScan Search identified a typical CAP domain (IPR014044) and a cysteine-rich secretory domain (PF00188) in *Dd*VAP2, classifying it as a member of the cysteine-rich secretory proteins, antigen 5, and pathogenesis-related 1 proteins (CAP) superfamily. A phylogenetic tree of *Dd*VAP2 was constructed with the identified VAPs from other plant parasitic nematodes, as well as all the homologues and paralogues of *Dd*VAP2 from different *D. destructor* isolates published on NCBI (**Figure 2**). The results showed that all the homologues of *Dd*VAP2 from different isolates of *D. destructor* clustered together in one clade. Interestingly, all VAPs from *D. destructor*, except for four VAPs (including *Dd*VAP1, AFQ55440.1), clustered in one clade with *Ha*VAP2. The four separated VAPs of *D. destructor* were clustered with the VAPs from *B. xylophilus*, another important migratory nematode. In addition, VAPs from different sedentary plant parasitic nematodes clustered together in one clade. This result indicates that the evolution of VAPs in *D. destructor* could have occurred through different origins.

### 3.2 The signal peptide of *Dd*VAP2 could lead protein secretion

**Figure 3:**
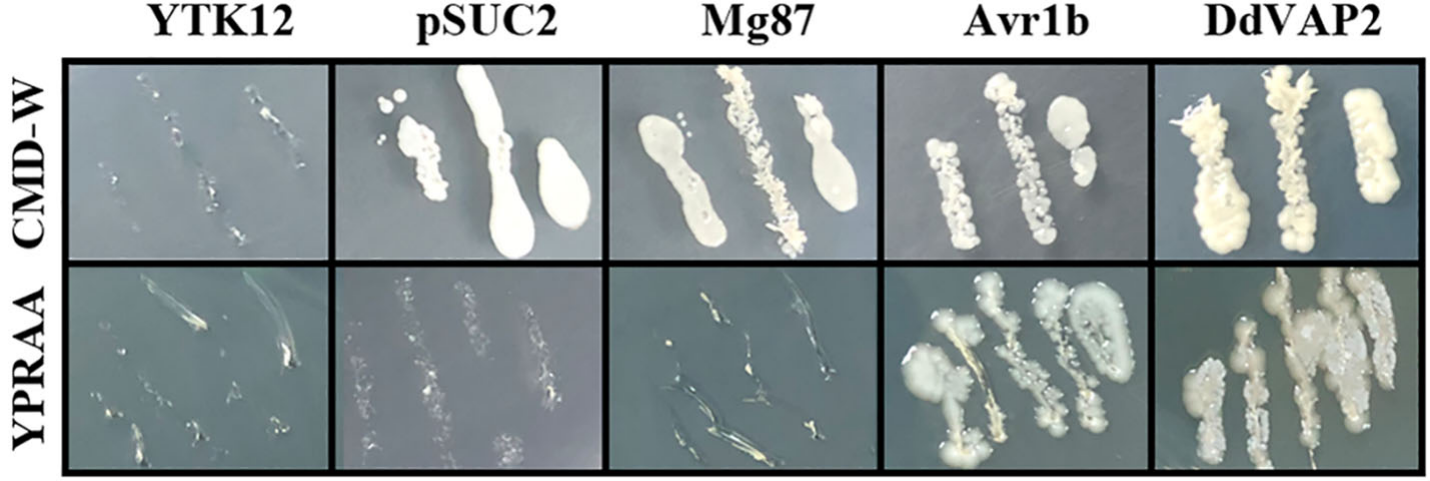
Functional validation of the signal peptide of *Dd*VAP2. YTK12 was untransformed *Saccharomyces cerevisiae* strain, and the YTK12 strain carrying the original pSUC2 vector were defined as pSUC2. The first 25 amino acids of Mg87 (no signal peptide function) from *Magnaporthe oryzae* was transformed into YTK12 strain with the pSUC2 vector to serve as a negative control. The signal peptides of Avr1b from *Phytophthora sojae* and *Dd*VAP2 were cloned into pSUC2 for expression in YTK12 strains. CMD-W is the medium with 0.67% yeast nitrogen base without amino acids, 0.075% tryptophan dropout supplement, 2% sucrose, 0.1% glucose and 2% agar. And YPRAA is the medium with 1% yeast extract, 2% peptone, 2% raffinose, 2mg/ml antimycin A and 2% agar.

To confirm the function of the signal peptide found in *Dd*VAP2, its signal peptide encoding sequence was functional validated using the yeast secretion system. As shown by the results, all the transformed YTK12 strains with either the original pSUC2 vector or the recombinant vectors, including the negative control, could grow on the CMD-W medium, while only the transformants containing the signal peptide of Avr1b or *Dd*VAP could grow on YPRAA medium (**Figure 3**). As the signal peptide of Avr1b was the positive control, which could lead the secretion of SUC2 for complementation the transformed yeast strain growth on medium YPRAA, it suggested that the signal peptide of *Dd*VAP2 had the same function of secretion. This result indicated that the signal peptide of *Dd*VAP2 could lead the protein secretion.

### 3.3 *Dd*VAP2 was highly expressed in the interaction between *D. destructor* and potato or sweet potato

**Figure 4:**
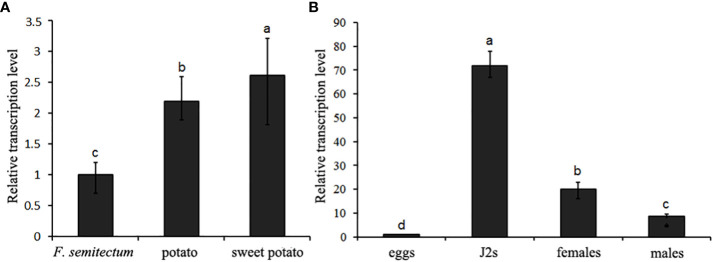
Transcription pattern of *DdVAP2* in *Ditylencus destructor*. **(A)** Relative expression levels of *DdVAP2* in (*D*) *destructor* populations cultured on either *F. semitectum*, potato or sweet potato. **(B)** Relative expression levels of *DdVAP2* in different life stages of *Ditylencus destructor*. Vertical lines indicate standard errors of the mean from two independent biological replicates. Different letters indicate significant differences (P < 0.05).

To clarify the transcription pattern of *DdVAP2* in *D. destructor*, its expression levels were first assessed in the three populations cultured on *F. semitectum*, potato and sweet potato, respectively. An up-regulation of *DdVAP2* with the expression levels of 2.2 ± 0.4 and 2.6 ± 0.5 fold was found on the populations of potato and sweet potato compared to the population on *F. semitectum*, respectively (**Figure 4A**). To further reveal the relative transcription levels of *DdVAP2* in different life stages, the transcription profiles of *DdVAP2* was examined from eggs, J2s, females and males collected from the population cultured on sweet potato, in which the transcription level of *DdVAP2* was the highest. As shown by the results, *DdVAP2* was highly expressed in all life stages except for eggs, with the highest expression level in J2s (**Figure 4B**). Additionally, higher expression level of *DdVAP2* was found in females than in males, with a significant difference (P<0.05). All the results demonstrate that *DdVAP2* is highly expressed in the infective stages of *D. destructor*, suggesting an important role of *DdVAP2* in *D. destructor* infection of plants.

### 3.4 *Dd*VAP2 was specifically expressed in the subventral esophageal glands

**Figure 5:**
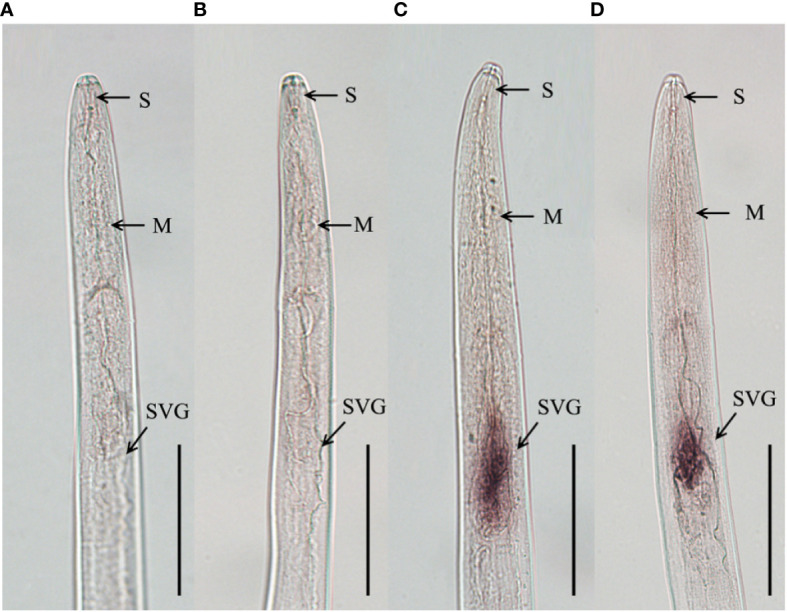
Tissue localization of *DdVAP2* in *Ditylenchus destructor*. **(A, B)** No hybridization signals were detected in negative controls with the digoxigenin-labeled sense *DdVAP2* RNA probe. **(C, D)** Specific hybridization signals were detected in the esophageal glands of *D. destructor* with the digoxigenin-labeled antisense *DdVAP2* RNA probe. S, stylet; M, median bulb; SVG, subventral oesophageal glands. Bars indicate 50 μm.

To clarify the tissue expression characteristics of *DdVAP2*, the *in situ* hybridization analysis was performed. Significant signals were observed in the subventral esophageal gland cells of the nematodes with the DIG-labelled antisense probes, but no signals was observed with the DIG-labelled sense probes (**Figure 5**). These results indicated that *DdVAP2* was specifically expressed in the subventral esophageal glands of *D. destructor*.

### 3.5 *Dd*VAP2 localized in both the cytosol and nucleus of plants

**Figure 6:**
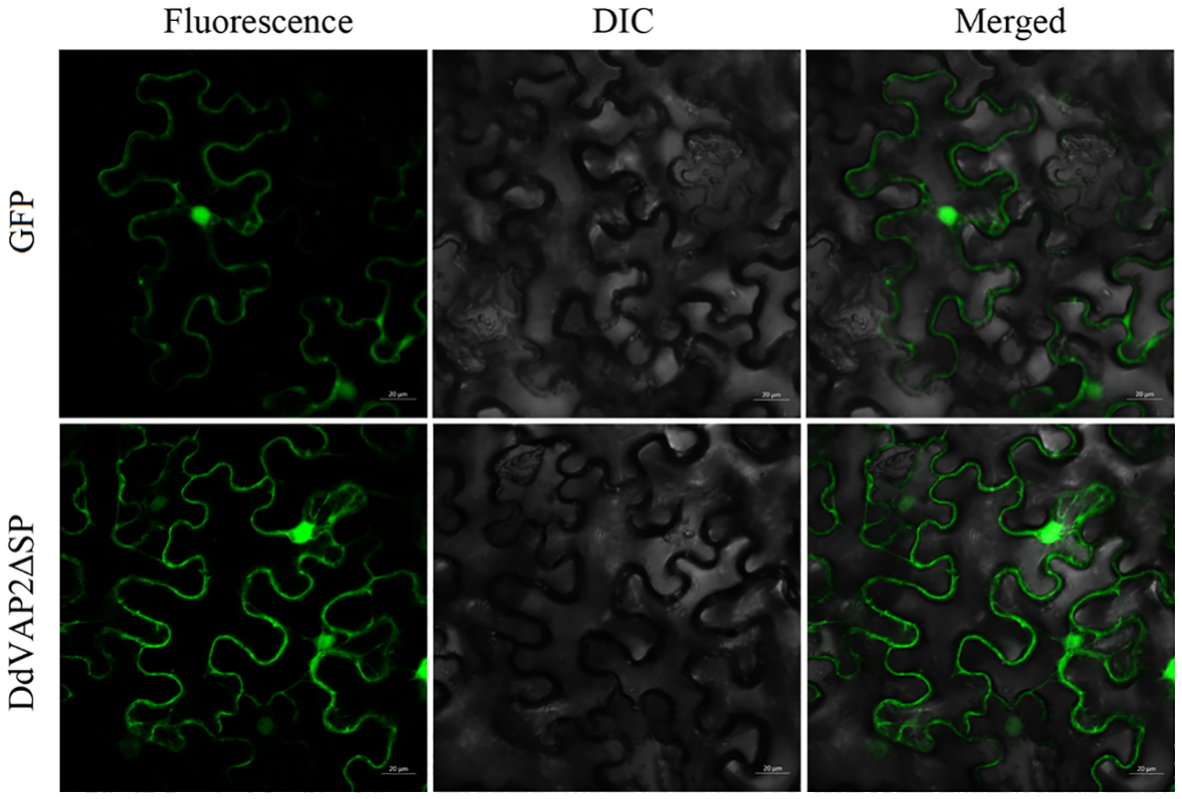
Subcellular localization of *Dd*VAP2 in *Nicotiana benthamiana*. The original vector expressing only green fluorescent protein (GFP) was control. The transient expression of a *Dd*VAP2ΔSP-GFP fusion protein was named as *Dd*VAP2ΔSP. DIC is light field, and Merged is the combination of both fluorescence and light field. Bars indicate 20 μm.

To investigate the subcellular localization of *Dd*VAP2 in plants, *Dd*VAP2 was heterologous expressed in *N. benthamiana* without its signal peptide, as the signal peptide would be cut off during secretion. The original vector expressing only green fluorescent protein (GFP) was used as a control. The fusion protein of *Dd*VAP2ΔSP-GFP was generated to assess the subcellular localization of *Dd*VAP2 in *N. benthamiana* accounting for cleavage of the signal peptide of *Dd*VAP2. The results demonstrated that the localization of *Dd*VAP2ΔSP-GFP was similar to the control, indicating the localization of *Dd*VAP2 in both the cytosol and nucleus in *N. benthamiana* as control (**Figure 6**).

### 3.6 Knocking down *DdVAP2* by RNAi impaired the infection and reproduction ability of *D. destructor* on plants

**Figure 7:**
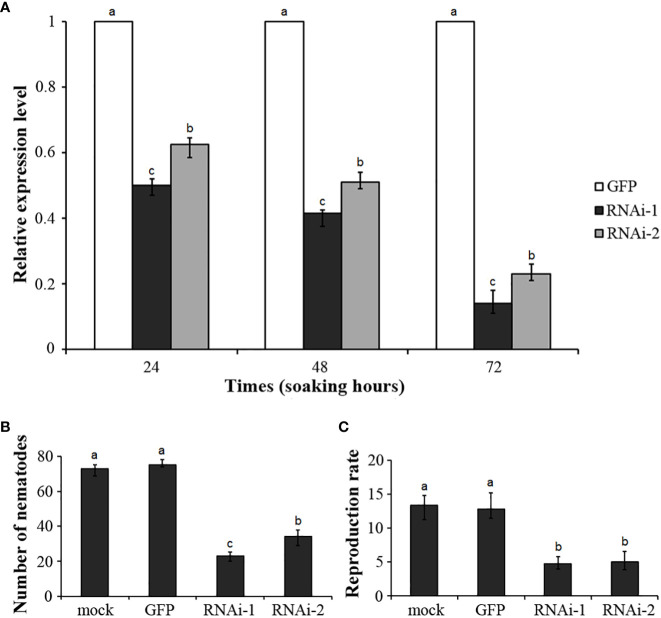
Functional analysis of *DdVAP2* during *Ditylenchus destructor* interaction with plants using RNAi. **(A)** Knock-down efficiencies of *DdVAP2* after nematodes soaked in soaking buffer containing dsRNA of either GFP, RNAi-1 or RNAi-2 for 24, 48 and 72 h. Values are expressed relative to the reference gene actin (GenBank accession no. GQ327968), with the dsRNA of GFP treatment set to 1. Values represent mean ± SE of three independent replicates. Differences were assessed using ANOVA. Different letters indicate P < 0.05. **(B, C)** Number of nematodes and reproduction rate in different treatments. All the statistical results were obtained from five independent replicates and values represent mean ± SE of three independent biological replicates. Differences were assessed using ANOVA. Different letters indicate P < 0.05.

To reveal the role of *DdVAP2* in parasitism by *D. destructor*, two independent RNAi fragments were designed to knock down this gene. Efficient stimulation of nematode soaking buffer ingestion was achieved using FITC as the marker (**Figure S2**). The expression levels of *DdVAP2* was sufficiently knocked down by both of the two RNAi fragments (RNAi-1 and RNAi-2) compared to the negative control, which was treated with dsRNA of GFP (GFP), according to our qRT-PCR analysis (**Figure 7A**). After incubation in the soaking buffer containing dsRNA of RNAi-1, RNAi-2 and GFP for 24 h, the transcription levels of *DdVAP2* were 50.16% and 62.66% in RNAi-1 and RNAi-2 treatments, compared to the GFP treatment. An increase in the knocking down efficiency was observed with longer incubating time. After 72 h of incubation, the transcription levels of *DdVAP2* were less than 20% in both the RNAi-1 and RNAi-2 treatments compared to the GFP treatment. These results suggest that significant suppression of the transcription of *DdVAP2* can be achieved with either RNAi-1 or RNAi-2 fragments. And the optimal gene-silencing effects can be achieved by treating the nematodes with either of the two RNAi fragments for 72 h in the soaking buffer.

Infection assays were conducted on both the seedlings and storage roots of sweet potato. The nematodes incubated in the soaking buffer without any dsRNA were used as mock control. Nematodes incubated in the soaking buffer containing dsRNA of RNAi-1, RNAi-2 or GFP for 72 h were used as the RNAi-1, RNAi-2 and GFP treatment, respectively. No significant change in survival rate was observed among nematodes in different treatments after 72 h incubation. After inoculation on sweet potato seedlings for 7 d, infected nematodes inside roots were 73 ± 3, 75 ± 2, 23 ± 2 and 34 ± 4 individuals in the mock, GFP, RNAi-1 and RNAi-2 treatments, respectively (**Figure 7B**). These results suggest that knocking down *DdVAP2* could significantly impair the infection ability of *D. destructor*. Subsequently, after inoculating treated nematodes on storage roots of sweet potato, the reproduction ability showed significant differences across different treatments. The reproduction rate was 13.4 ± 1.7 in mock, 12.8 ± 1.8 in GFP, 4.7 ± 0.9 in RNAi-1 and 5.0 ± 1.4 in RNAi-2, respectively (**Figure 7C**).

## 4 Discussion

Understanding the molecular mechanisms underlying plant-nematode interactions is crucial for developing novel strategies to control plant parasitic nematodes (Eves-Van Den Akker et al., 2021; Abd-Elgawad, 2022). In recent years, research on cloning and functional characterization of parasitism genes, especially effectors, from plant parasitic nematodes has attracted significant interest (Vieira and Gleason, 2019; Goverse and Mitchum, 2022). However, our current knowledge in this field primarily comes from sedentary endoparasitic plant nematodes (Rutter et al., 2022). *D. destructor*, as a severe destructive plant parasitic nematode that poses a significant threat to various root and tuber crops, requires extensive research to uncover its molecular pathogenic mechanism. In this study, we focused on functional analysis of one effector conserved in both sedentary and migratory plant parasitic nematodes, VAP, from this important migratory endoparasitic plant nematode.

In our study, we cloned the ORF of *DdVAP2* from the Xi’an isolate of *D. destructor*. Eight nucleotide substitutions compared to the homologue from the EST library of *D. destructor* constructed with the isolate from Tongshan, Jiangsu, China were observed (Peng et al., 2013), and these substitutions were predicted to influence the protein function by SIFT4G analysis. Phylogenetic analysis revealed that there is a significant genetic diversity of VAPs from different *D. destructor* populations, as much more homologues of *Dd*VAP2 were found to cluster with these two identified VAPs from *D. destructor* in the same clade. This suggests a strong variation among VAPs from different *D. destructor* populations. This diversity could be attributed to SNP variations among different isolates of *D. destructor*, as it has been reported that high intraspecific polymorphism within different populations of *D. destructor* (Huang et al., 2010). The phylogenetic analysis also showed that the clade in which *Dd*VAP2 clustered contained a well-characterized VAP from *H. avenae*, *Ha*VAP2. It suggests a possible common origin between *Dd*VAP2 and *Ha*VAP2, indicating potential functional similarities between them. This differentiation from other VAPs identified from sedentary plant nematodes is noteworthy. Furthermore, four VAPs of *D. destructor* were found to cluster with three VAPs identified from the important migratory plant parasitic nematode, *B. xylophilus*. This suggests that the VAPs of *D. destructo*r have evolved from different origins, which is in accordance with the previous finding that VAPs could have divergent origins in helminths (Wilbers et al., 2018).

As *D. destructor* can feed and reproduce on both plants and fungus, the transcription profile of *DdVAP2* was compared between the populations cultured on potato, sweet potato and *F. semitectum*. The transcription levels of *DdVAP2* were significantly higher in populations cultured on potato and sweet potato compared to those cultured on fungus. This suggests that the expression of *DdVAP2* is highly induced during the interaction with plants. This result is in consistent with the expression patterns of VAPs from *B. xylophilus* (Kang et al., 2009). Although the developmental expression patterns of the three VAPs in *B. xylophilus* showed no significant differences between different life stages (eggs, hatched second-stage juveniles, adult females and males) (Lin et al., 2011), *DdVAP2* was highly expressed in all infective stages when interacting with sweet potato, which means all the life stages except for eggs. This expression pattern is similar to that of *RsVAP*, the VAP from another migratory nematode *R. similis*, which showed a significantly higher expression level in juveniles and females to facilitate the nematode infection (Li et al., 2021). The absence of *RsVAP* expression in males of *R. similis* was a result of their inability to infect plants. Therefore, it could be concluded that both *DdVAP2* and *RsVAP* play important roles in the successful infection of migratory nematode infection on plants. In *M. incognita*, *Mi-vap-2* was highly expressed in pre-parasitic J2 and early-satge juveniles at 2 or 7 days post-inoculation (dpi), but it was hardly detected in eggs, 13-dpi juveniles or adult females during the interaction with tomato. It suggests an important role of VAP in the early stage of parasitism (Wang et al., 2007). Similarly, in the sedentary nematode *G. rostochiensis*, Gr-VAP1 was highly expressed in migratory stages of nematodes, while the expression level declined or was even absent in the sedentary stages (Lozano-Torres et al., 2014). Taken together, these findings indicate that the expression of VAPs is closely related to plant nematode migration inside hosts to establish parasitism. However, in a study of VAPs from *H. avenae*, another important sedentary nematode, two *HaVAP*s showed distinct expression patterns (Luo et al., 2019). The expression pattern of *HaVAP1* was in accordance with the above conclusion, but *HaVAP2* was highly expressed in the late parasitic stages, such as J3, J4 and females. This result indicated that VAPs could play functions not only in the onset of parasitism, but also in the maintenance of parasitism. As *Dd*VAP2 is clustered with *Ha*VAP2 in the same clade, we speculated that *DdVAP2* could also play important roles in both establishing and maintaining the parasitism on plants.

As reported, VAPs are abundantly secreted by helminths for parasitism in both plant-parasitic and animal-parasitic nematodes (Wilbers et al., 2018). In our study, combining the functional validation of the signal peptide found in *Dd*VAP2 and the subventral esophageal glands-specific tissue localization of *DdVAP2*, it could be concluded that *Dd*VAP2 would be secreted into plants during the interaction between *D. destructor* and plants. To further clarify the function site of *Dd*VAP2 after secretion into plants, the subcellular localization of *Dd*VAP2 in *N. benthamiana* without its signal peptide was conducted. We found that *Dd*VAP2 minus signal peptide is localized in both the cytosol and nuclei of plant cells, differing from previous knowledge of sedentary nematode VAPs. In *G. rostochiensis*, Gr-VAP1 was considered to localize in the apoplast, where it could be recognized by the cell surface-localized receptors of plants (Lozano-Torres et al., 2014). In *H. avenae*, *Ha*VAP1 and *Ha*VAP2 were reported to localize in the chloroplasts and nucleus, respectively. Up to now, all the characterized plant nematode VAPs showed different subcellular localizations in plants, suggesting the diverse function mechanisms in ascending plant parasitic nematode infections. Both of *Ha*VAP2 and *Dd*VAP2 could be transported into the nucleus of plant cells without nuclear localization signals, as both of them clustered together in the same clade of phylogenetic tree. However, unlike all the reported VAPs of sedentary plant parasitic nematodes, *Dd*VAP2 could also localize in the cytosol of plants, suggesting it may have a distinct interaction target compared to the sedentary plant parasitic nematode VAPs.

Finally, we used RNAi, which is a powerful tool for functional analysis of nematode genes, to demonstrate the function of *DdVAP2* in the interaction between *D. destructor* and plants. Our findings show that knocking down the expression of *DdVAP2* significantly impaired the infection and reproduction ability of *D. destructor* on plants. The results showed that *DdVAP2* was essential for the successful infection and reproduction of *D. destructor* on plants, which is in consistent with the important role of VAPs in parasitism found in other plant parasitic nematodes, as their virulence is impaired when VAPs are silenced (Han et al., 2023). In root-knot nematodes, silencing *Mg-VAP1* reduced the ability of *M. graminicola* to colonize rice plant roots (Petitot et al., 2020), and silencing *Mhi-vap-1* significantly reduced *M. hispanica* attraction to roots, penetration, development and reproduction in tomato plants (Duarte et al., 2017). Additionally, up-regulated expression of *Mi-vap-2* was found to promote nematode pathogenicity in progeny (Chi et al., 2016). In cyst nematodes, VAPs identified from both *H. schachtii* and *H. avenae* were found to suppress plant basal immunity (Lozano-Torres et al., 2014; Luo et al., 2019). In *R. similis*, another important migratory endoparasitic nematode, silencing *RsVAP* attenuated the nematode pathogenicity (Li et al., 2021). These findings collectively suggest that VAPs play a facilitative role in nematode parasitism as observed in our study. However, in case of *H. avenae*, knocking down *Ha*VAP1 by RNAi promoted its parasitism on susceptible barley, suggesting an avirulent function of this VAP (Luo et al., 2019). We speculated that this could be a result of the plant recognition of this important pathogenic effector, leading to the induction of plant defense responses, similar to the situation of Gr-VAP1 recognized by plants (Lozano-Torres et al., 2012; Lozano-Torres et al., 2014). This implies that some plants have evolved receptors to recognize these important and conserved effectors during the long coevolution with plant parasitic nematodes.

## 5 Conclusion

In this study, a variant of the venom allergen-like protein gene, *DdVAP2*, was cloned from *D. destructor*. Our findings revealed that *DdVAP2* is highly expressed and secreted into plants during the interaction between *D. destructor* and plant. This suggests that DdVAP2 plays a crucial role in facilitating the nematode infection. It also provides a new insight into the molecular pathogenic mechanism of *D. destructor*. The subcellular localization of *Dd*VAP2 in both the cytosol and nucleus of plant cells indicates a specific molecular pathogenic mechanism employed by *D. destructor*, which requires further research.

## Data availability statement

The datasets presented in this study can be found in online repositories. The names of the repository/repositories and accession number(s) can be found in the article/**Supplementary Material**.

## Author contributions

QC: Conceptualization, Data curation, Funding acquisition, Investigation, Methodology, Validation, Writing – original draft, Writing – review & editing. YY: Data curation, Investigation, Validation, Writing – original draft. BH: Data curation, Investigation, Validation, Writing – original draft. YZ: Data curation, Investigation, Validation, Writing – original draft. MZ: Data curation, Investigation, Validation, Writing – original draft. SH: Writing – original draft. FZ: Supervision, Writing – review & editing. HP: Supervision, Writing – review & editing. DP: Funding acquisition, Methodology, Supervision, Writing – review & editing. YL: Conceptualization, Supervision, Writing – review & editing.
