# Supplementary figures and images for: A variant of the venom allergen-like protein, DdVAP2, is required for the migratory endoparasitic plant nematode Ditylenchus destructor parasitism of plants
Source: Front Plant Sci. 2023 Dec 13;14:1322902. doi: 10.3389/fpls.2023.1322902 (PMC10751354; doi:10.3389/fpls.2023.1322902)

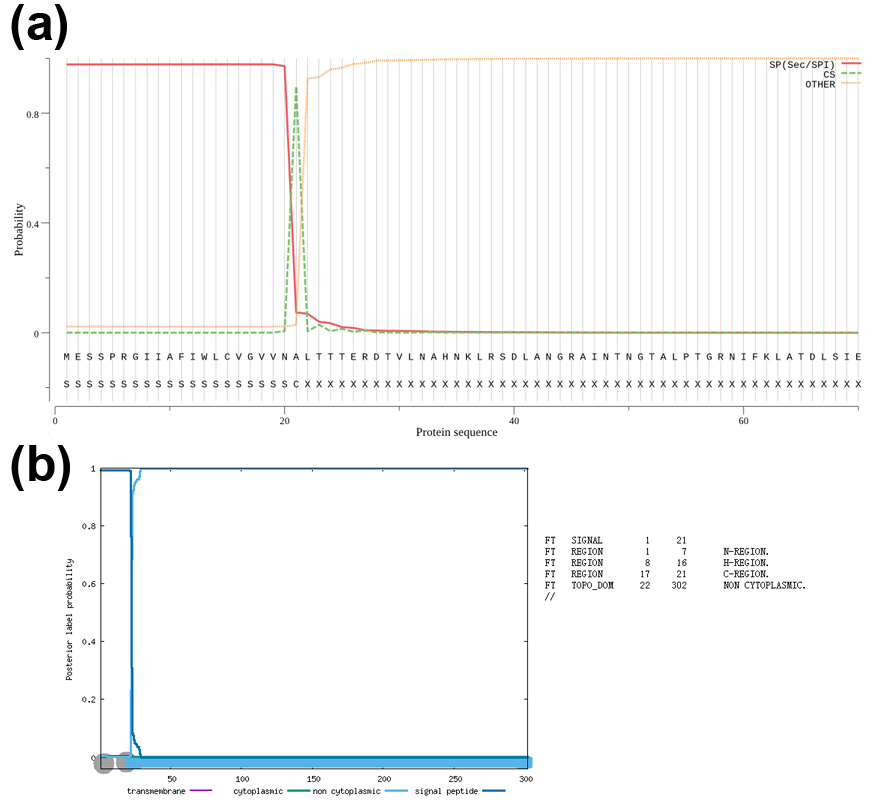

Supplement: Supplementary file 1 [file Image_1.tif]

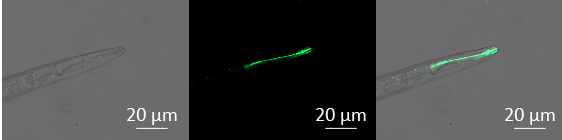

Supplement: Supplementary file 2 [file Image_2.tif]
