# Supplementary material for: A variant of the venom allergen-like protein, DdVAP2, is required for the migratory endoparasitic plant nematode Ditylenchus destructor parasitism of plants
Source: Front Plant Sci. 2023 Dec 13;14:1322902. doi: 10.3389/fpls.2023.1322902 (PMC10751354; doi:10.3389/fpls.2023.1322902)
Supplement: Supplementary file 3 [file Table_1.docx]

**Table S1. The primers used in this study**

| **Primer** | **Sequence** |
| --- | --- |
| **FL-F** | **ATGCTCACTACTACTGAACGGGA** |
| **FL-R** | **GCCGCTCGTCACAGAGCA** |
| **SP-F** | **ATGCTCACTACTACTGAACGGGA** |
| **SP-R** | **TGCATTCACAACACCAACACA** |
| **qRTPCR-F** | **GCCGAGCGAAATAATACAG** |
| **qRTPCR-R** | **GCCAATGTGGTCGTAACTG** |
| **Probe1-F** | **GTGGTGCCCAACTCAAG** |
| **Probe1-R** | **CCAATGTGGTCGTAACTG** |
| **Probe2-F** | **GGCAACCGACCTTTCAAT** |
| **Probe2-R** | **ACTCAGTCTGGGTCAAG** |
| **SL-F** | **CGGGATCCATGCTCACTACTACTGAACGGGA** |
| **SL-R** | **GCGTCGACGCCGCTCGTCACAGAGCA** |
| **RNAi-1F** | **TAATACGACTCACTATAGGGTGGAATCTTCTCCTCGAGGA** |
| **RNAi-1R** | **TAATACGACTCACTATAGGGTCAGCGCTTCAACAAAGTTTT** |
| **RNAi-2F** | **TAATACGACTCACTATAGGGACTCAAGGATTCACCATTGTA** |
| **RNAi-2R** | **TAATACGACTCACTATAGGGGAAGTGACACTCACTTTGCAG** |
